# Prevalence of re-laparotomy and its risk factors in patients who underwent gastrointestinal procedure at Referral Hospital in Ethiopia

**DOI:** 10.1371/journal.pone.0335304

**Published:** 2026-05-29

**Authors:** Kumlachew Geta, Amsalu Molla, Yonas Kassaw, Simegnew Kibret, Metages Hunie, Kaletsidk Dessalegn, Diriba Teshome, Belayneh Dessie, Keder Essa, Efrem Fenta

**Affiliations:** 1 Department of Anesthesia, College of Health Sciences, Debre Tabor University, Debre Tabor, Ethiopia; 2 Department of Surgery, College of Health Sciences, Debre Tabor University, Debre Tabor, Ethiopia; 3 Departement of Emergency and critical care Medicine, College of Health Sciences, Debre Tabor University, Debre Tabor, Ethiopia; Public Library of Science, UNITED STATES OF AMERICA

## Abstract

**Background:**

Re-laparotomy is one of the causes of morbidity and mortality among patients who have had abdominal surgery. Due to the unavailability of laparoscopic surgery in the region, laparotomy remains the standard treatment. As a result, re-laparotomy is commonly performed, significantly increasing the labor load, financial costs, and the risks of morbidity and mortality for patients. The aim of this study is to evaluate the prevalence and factors associated with re-laparotomy procedures, in order to better understand their impact on patient health and healthcare resources.

**Methods:**

A retrospective cross-sectional study was conducted on patients who underwent gastrointestinal procedures in a hospital in Ethiopia between September 2020 and August 2022. The association between independent variables with re-laparotomy (dependent variable) was analyzed using bi-variate and multivariate logistic regression. Those variables that had a p-value of less than 0.2 were put into a multivariate analysis. The strength of the association was displayed using crude and adjusted odds ratios with a 95% confidence interval. P-values less than 0.05 were used to determine statistical significance.

**Results:**

A total of 1276 charts were reviewed, and 127 (10%) of those were found to have re-laparotomy. A greater proportion of re-laparotomy was associated with the occurrence of prior abdominal surgery (AOR = 67.94, 95% CI: 39.07, 118.13), the existence of ischemic bowel during surgery (AOR = 4.36, 95% CI: 2.10, 9.02), and the intraoperative use of inotropic/vasopressor medications (AOR = 7.03, 95% CI: 2.52, 19.59).

**Conclusions:**

The prevalence of re-laparotomy was found to be 10% which is higher compared with some previous reports. Prior abdominal surgery, ischemic bowel presence during surgery, and the use of inotropic/vasopressor drugs intra-operatively were all found to be risk factors for re-laparotomy.

## Introduction

Re-laparotomy is a re-abdominal ‌‌operation performed within 60 days of the first surgery [1]. According to studies from various regions of the world, it represents between 1.5 and 27% of patients undergoing abdominal surgery [2–5]. The rate of re-laparotomy following gastrointestinal surgery is a significant surgical workload both globally and in the Ethiopian context. Internationally, the rate of re-laparotomy ranges from 1.5% to 27% [6], the Ethiopian figure of 6.8% [7] being a significant percentage of the surgical workload in the developing world. The most frequent reasons for reoperation are intra-abdominal collection and anastomotic leakage are worldwide [8], although their prevalence and outcome are remarkably variable worldwide. Peritonitis, hemorrhage, abscess, wound dehiscence, necrotizing pancreatitis, intestinal necrosis, bowel obstruction, and evisceration are some of the common causes of re-laparotomy [9–11].

Patients who have re-laparotomies experience higher medical expenses [12,13] who suffer from higher morbidity (surgical site infection, wound dehiscence, hospital-acquired infections, etc.) and increased mortality [14–16].

Even though preoperative and postoperative antibiotics, counting of instruments, processing of instruments, and wound care are used as preventive measures, issues with the health system’s service, and patient factors [9,17,18] may be the underlying causes associated with poorer outcomes following the initial surgery [19–21].

The mortality of re-laparotomy rates in Ethiopia is ranging from 12.8% [7] to 26.4% [22]. These statistics underscore the need to continue researching this subject. It is critical to address identification of risk factors, optimization of the timing of intervention, and development of management protocols contextualized to the host environment. These are to be addressed to close knowledge gaps, identify high-risk groups, and maximize resource utilization, especially in low-resource settings like Ethiopia. Currently, there is no sufficient data on this issue in the country. This study, therefore, aims to determine the prevalence of re-laparotomy and its risk factors at a referral hospital in Ethiopia.

### Objective

#### Main objective.

To assess the prevalence of re-laparotomy and its risk factors in patients who underwent gastrointestinal procedures: A retrospective cross-sectional study.

#### Specific objective.

To determine the prevalence of re-laparotomy among patients who underwent gastrointestinal procedures.

To describe risk factors that lead to re laparotomy among patients who underwent gastrointestinal procedures.

## Methods

### Study design, period and setting

Debre Tabor Comprehensive Specialized Hospital (DTCSH) is a prominent referral center in the South Gondar Zone of the Amhara region in Ethiopia, providing a wide range of surgical, diagnostic, and inpatient services with on gastrointestinal surgery. This makes the hospital an essential source of comprehensive data for the study. Its well-organized and relatively complete medical records system ensures the accuracy and reliability of this retrospective design. Furthermore, the hospital handled a high volume of gastrointestinal surgeries and addressed a diverse range of cases during the study period from September 2020 to August 2022. This study is registered at research registry.com with the number researchregistry10331.

### Inclusion criteria/Exclusion criteria

The study included the records of adult patients (≥ 18 years of age) who underwent gastrointestinal surgery, were admitted to DTCSH during the study period, and whose charts contained all necessary information. Patients’ charts that underwent the first surgery but afterward had referrals to other facilities were excluded.

### Sampling technique

A survey of all patient records for people who had laparotomy or gastrointestinal surgeries using their medical identification numbers.

### Variables of the study

#### Dependent variable.

Re-laparotomy (Yes/No).

### Independent variables

Demographic characteristics of patients: age, sex, residence, American Society of Anesthesiologists (ASA) physical status, presence of coexisting disease, Duration of present illness; and Anesthesia and surgery-related characteristics of patients: The urgency of surgery, type of operator, site of surgery, antibiotics prophylaxis, administration of the inotropic or vasopressor agent, Presence of ischemic bowel intraoperatively, presence of malignancy, duration of surgery, and duration of anesthesia.

### Data collection technique

A structured checklist which is adapted from previous studies was used to collect the data. Patients’ medical registration numbers were noted in the operating room record books. Using card numbers as the primary identifier, the charts of patients were registered from the chart room, and the total 2-year of laparotomy from September 2020 to August 2022 was counted. By providing training and the proper supervision to data collectors who are 3 BSc. Anesthesia providers, data quality was controlled. The instrument’s validation was carried out through a pilot to produce Cronbach’s alpha results.

### Data entry and analysis

The data was coded and entered into SPSS version 22. Association of independent variables with outcome variable were analyzed by using binary logistic regression with the dependent variable and those with a p-value of ≤ 0.2 from the bi-variate analysis were fitted to a multivariate logistic analysis to check their association with the outcome variable. Adjusted odds ratios with 95% CI were used to show the strength of the association. P-values of < 0.05 were considered statistically significant. The finding of this research was reported according to STROBE guideline (S1 Checklist).

### Ethics statement

Debre Tabor University, college of health sciences ethical review committee granted its ethical approval. Additionally, permission to carry out the research was secured from the hospital’s administrative office. To maintain confidentiality, data was kept anonymous in the distributed data extraction format.

## Results

### Demographic characteristics of patients

The study included 1276 individuals in total who had gastrointestinal surgery performed under general anesthesia. Rural areas accounted for 71% of the patient population. Of the participants in this research, 84.6% had ASA physical status I and 98.1% had no history of co-existing diseases (S1 Table).

### Anesthesia and surgery-related characteristics of patients

Seven hundred seventy-one patients (60.4%) had undergone emergency surgery. The large bowel was the second most common site of surgery, with 403 (31.6%) patients having appendectomy (467, or 36.6%), respectively. Before surgery, prophylactic antibiotic was given to 1026 (80.4%) of the patients (S2 Table).

### The prevalence of re-laparotomy

Re-laparotomy was performed on 127 (10%) of the 1276 surgical patients. A total of 34 (2.7%) patients experienced intraoperative complications. While 1264 (99.1%) of the patients were discharged with improved outcomes, 12 of them were dead. Infection at the site of the previous operation was the most frequent reason for re-laparotomy 56 (4.4%), and in 36 (2.8%) of the patients, the anastomosis was lavaged and sutured.

### Factors associated with re-laparotomy

Categorical variables were evaluated to ensure that chi-square test assumptions were met and were examined for potential associations. Additionally, variables identified as clinically significant were included directly in the bivariate regression analysis.

Among the variables assessed in the bivariate logistic regression analysis, seven demonstrated statistical significance (P-value ≤ 0.2). This included sex, urgency of the procedure, history of previous abdominal surgery, ischemic bowel, use of intraoperative vasopressors, type of operator, and surgical site. Accordingly, these variables were incorporated into the multivariate logistic regression model for further analysis.

In multivariate logistic regression, there was a statistically significant association between re-laparotomy and intraoperative administration of inotropic/vasopressor drugs, presence of ischemic bowel, and previous abdominal surgery. Re-laparotomy incidence was substantially associated with previous abdominal surgery (AOR = 67.94, 95% CI: 39.07, 118.13) and the existence of ischemic bowel (AOR = 4.36, 95% CI: 2.10, 9.02). This study also demonstrated that a higher incidence of re-laparotomy was significantly associated with the intraoperative administration of inotropic/vasopressor drugs (AOR = 7.03, 95% CI: 2.52, 19.59). (S3 Table).

## Discussion

Re-laparotomy lengthens the hospital stay, increases the risk of infection, and in a substantial number of patients, and even causes death. Therefore, re-laparotomy following gastrointestinal procedures may significantly increase patient morbidity and mortality [23–28]. In this study, 127 had performed re-laparotomy out of 1276 patients who underwent laparotomy with an overall prevalence of 10% (95% CI 8.354, 11.646).

Similar results were found by Birhanu et al. in Ethiopia, who found that 48 patients who underwent re-laparotomy had done so, with a prevalence of 12.3% overall among the 390 patients who had undergone laparotomy [6]. This proximity in results may be attributable to the similarity in the level of the hospitals where the research was conducted. Another study by Reddy et al. reported that in a total of 520 consecutive patients who underwent pancreaticoduodenectomy, 96 patients (18.5%) underwent a relaparotomy [29]. This increased outcome might be attributed to the crucial nature of the whiples surgery (pancreaticoduodenectomy), which renders relaparotomy more common. In a prospective cohort study of patients undergoing elective abdominal surgery, Strik et al. found that 160 (27%) of the 604 patients needed repeat abdominal surgery. According to Kim et al., 29 (22% of the 129 patients who underwent emergency surgery for non-trauma intra-abdominal catastrophes required relaparotomy [30]. The study’s focus on elective and emergency gastrointestinal surgery may explain the disparity. However, the other studies mainly covered emergency procedures, which raises the risk of complications and re-laparotomy prevalence.

Re-laparotomy incidence was substantially associated with previous abdominal surgery (AOR = 67.94, 95% CI: 39.07, 118.13) and the existence of ischemic bowel (AOR = 4.36, 95% CI: 2.10, 9.02). This study also demonstrated that a higher incidence of re-laparotomy was significantly associated with the intraoperative administration of inotropic/vasopressor drugs (AOR = 7.03, 95% CI: 2.52, 19.59). Similarly, A retrospective chart analysis done in Houston, USA, by Kim et al. revealed that the likelihood of relaparotomy was significantly associated with the presence of ischemic bowel (P = 0.02) [30]. A study by Kong et al. from South Africa revealed that 406 re-laparotomies were required in a total of 1000 patients with severe intra-abdominal infection related to complex appendicitis. Patients referred from any rural center, illness lasting more than five days, heart rate greater than 120 beats per minute, and perforation linked to generalized intra-abdominal sepsis were the factors predicting the need for a subsequent re-laparotomy [31]. A limitation of this study is its use of a heterogeneous population and the fact that it was single-centered, primarily due to financial constraints. However, strength of the study is the larger sample size used for the cross-sectional analysis.

## Conclusions

The overall prevalence of re-laparotomy in patients undergoing gastrointestinal surgeries was found to be 10% which is higher compared with some previous reports. Previous abdominal surgery, the presence of ischemic bowel and intraoperative administration of inotropic/vasopressor drugs were the factors that increase the risk of re-laparotomy. We recommend meticulous perioperative management for high-risk patients, particularly those with a history of abdominal surgery, bowel ischemia, or vasopressor use. Implementing risk-based protocols and promoting early, multidisciplinary interventions may help reduce re-laparotomy rates. Furthermore, additional multicenter prospective studies are necessary to validate these findings and guide the development of targeted interventions for clinical practice.

## Supporting information

S1 Checklist STROBE guideline. (DOCX)

S1 Table Demographic characteristics of patients who underwent gastrointestinal surgery at Debre Tabor Comprehensive Specialized Hospital (N = 1276). (DOCX)

S2 Table Anesthetic and surgery-related characteristics of patients who underwent gastrointestinal surgery at Debre Tabor Comprehensive Specialized Hospital (N = 1276). (DOCX)

S3 Table Factors associated with re-laparotomy patients who underwent gastrointestinal surgery at Debre Tabor Comprehensive Specialized Hospital (N = 1276). (DOCX)

## Abbreviations

AOR: Adjusted Odds Ratio
ASA: American society of anesthesiologists
CI: Confidence Interval
COR: Crude Odds Ratio
DTCSH: Debre Tabor Comprehensive Specialized Hospital
IESO: Integrated Emergency Surgical and Obstetric Officer
SPSS: Statistical package for Social science
