# Supplementary material for: Prevalence of re-laparotomy and its risk factors in patients who underwent gastrointestinal procedure at Referral Hospital in Ethiopia
Source: PLoS One. 2026 May 29;21(5):e0335304. doi: 10.1371/journal.pone.0335304 (PMC13220992; doi:10.1371/journal.pone.0335304)
Supplement: S1 Table — (DOCX) [file pone.0335304.s002.docx]

Table 1: Demographic characteristics of patients who underwent gastrointestinal surgery at Debre Tabor Comprehensive Specialized Hospital (N=1276).

| Variable | | Re-laparotomy. | | Total, Frequency (%) |
| --- | --- | --- | --- | --- |
|  |  | No | Yes |  |
| Age (in mean ± SD) * | | 33.6 ± 16.2 | 35.8 ± 15.6 |  |
| Sex | Male | 685 | 103 | 788 (61.8) |
|  | Female | 464 | 24 | 488 (38.2) |
| Residence | Urban | 337 | 34 | 371 (29.1) |
|  | Rural | 812 | 93 | 905 (70.9) |
| ASA status | ASA 1 | 975 | 104 | 1079 (84.6) |
|  | ASA 2 | 174 | 23 | 197 (15.4) |
| History of Co-existing disease | No | 1125 | 127 | 1252 (98.1) |
|  | Yes | 24 | 0 | 24 (1.9) |
| Duration of presenting illness in hours* | | 80 ± 89 | 47 ± 22 |  |

Note: * Expressed in mean ± SD, and analyzed by student T Test.
