# Supplementary material for: Prevalence of re-laparotomy and its risk factors in patients who underwent gastrointestinal procedure at Referral Hospital in Ethiopia
Source: PLoS One. 2026 May 29;21(5):e0335304. doi: 10.1371/journal.pone.0335304 (PMC13220992; doi:10.1371/journal.pone.0335304)
Supplement: S2 Table — (DOCX) [file pone.0335304.s003.docx]

Table 2: Anesthetic and surgery-related characteristics of patients who underwent gastrointestinal surgery at Debre Tabor Comprehensive Specialized Hospital (N=1276).

| Variable | | Re-laparotomy. | | Frequency (%) |
| --- | --- | --- | --- | --- |
|  |  | No | Yes |  |
| Urgency of first surgery | Elective | 483 | 22 | 505 (39.6) |
|  | Emergency | 666 | 105 | 771 (60.4) |
| Site of surgery | Pylorus | 45 | 0 | 45 (3.5) |
|  | Gall bladder | 138 | 0 | 138(10.8) |
|  | Small bowel | 176 | 47 | 223(17.5) |
|  | Appendix | 456 | 11 | 467(36.6) |
|  | Large bowel | 334 | 69 | 403(31.6) |
| Antibiotic treatment before surgery | No | 227 | 23 | 250 (19.6) |
|  | Yes | 922 | 104 | 1026 (80.4) |
| History of abdominal surgery | No | 1090 | 22 | 1112 (87.1) |
|  | Yes | 59 | 105 | 164 (12.9) |
| Presence of ischemic bowel | No | 1068 | 81 | 1149 (90.0) |
|  | Yes | 81 | 46 | 127 (10.0) |
| Administration of inotropic or vasopressor agents | No | 1126 | 92 | 1218 (95.5) |
|  | Yes | 23 | 35 | 58 (4.5) |
| Presence of malignancy | No | 1149 | 115 | 1264 (99.1) |
|  | Yes | 10 | 2 | 12 (0.9) |
| Duration of surgery in minutes* | | 97 ± 38 | 111 ± 52 |  |
| Duration of anesthesia in minutes* | | 105 ± 39 | 123 ± 54 |  |
| Type of operator | General surgeon | 761 | 82 | 843 (66.1) |
|  | IESO | 35 | 0 | 35 (2.7) |
|  | General Surgeon & IESO | 353 | 45 | 398 (31.2) |

Note: * Expressed in mean ± SD, and analyzed by student T-test.

IESO (integrated emergency surgical and obstetric officer)
