# Supplementary material for: Prevalence of re-laparotomy and its risk factors in patients who underwent gastrointestinal procedure at Referral Hospital in Ethiopia
Source: PLoS One. 2026 May 29;21(5):e0335304. doi: 10.1371/journal.pone.0335304 (PMC13220992; doi:10.1371/journal.pone.0335304)
Supplement: S3 Table — (DOCX) [file pone.0335304.s004.docx]

Table3: Factors associated with re-laparotomy patients who underwent gastrointestinal surgery at Debre Tabor Comprehensive Specialized Hospital (N=1276).

| **Variable** | **Characteristics** | **COR (95% CI)** | **AOR (95% CI)** | **P-value** |
| --- | --- | --- | --- | --- |
| Sex | Male | 2.06 (1.29–3.29) * | 1.48 (0.89–2.45) | 0.13 |
|  | female | 1 | 1 | - |
| Urgency of the procedure | Emergency | 3.42 (2.11–5.56) * | 1.80 (0.95–3.42) | 0.07 |
|  | Elective | 1 | 1 | - |
| Type of operators | IESO | 0.05 (0.00–0.94) * | 0.45 (0.10–2.00) | 0.30 |
|  | General surgeon and IESO | 1.23 (0.84–1.80) | 1.17 (0.77–1.77) | 0.46 |
|  | General surgeon | 1 | 1 | - |
| Site of surgery | Pylorus | 0.06 (0.00–1.00) | 0.09 (0.01–1.12) | 0.06 |
|  | Gall bladder | 0.05 (0.00–0.87) * | 0.45 (0.10–2.00) | 0.16 |
|  | Small bowel | 1.28 (0.85–1.92) | 1.13 (0.70–1.83) | 0.61 |
|  | Appendix | 0.67 (0.35–1.26) | 0.64 (0.32–1.28) | 0.20 |
|  | Large bowel | 1 | 1 | - |
| Previous abdominal surgery | Yes | 88.17(51.95, 149.65) * | 67.94(39.07, 118.13) | < 0.001** |
|  | No | 1 | 1 | - |
| Presence of ischemic bowel | Yes | 7.49(4.89, 11.47) * | 4.36 (2.10, 9.02) | < 0.001** |
|  | No | 1 | 1 | - |
| Intraoperative administration of inotropic/vasopressor agents | Yes | 18.63(10.56, 32.85) * | 7.03(2.52, 19.59) | < 0.001** |
|  | No | 1 |  | - |

* Associated in bivariate analysis ** associated in multivariate analysis
